# PF-05231023, a long-acting FGF21 analogue, decreases body weight by reduction of food intake in non-human primates

**DOI:** 10.1007/s10928-016-9481-1

**Published:** 2016-07-12

**Authors:** W. Clayton Thompson, Yingjiang Zhou, Saswata Talukdar, Cynthia J. Musante

**Affiliations:** Pfizer Inc, 610 Main Street, South Bldg, 4th Floor, Cambridge, MA 02139 USA; Merck Research Laboratories, 33 Avenue Louis Pasteur, Boston, MA 02115 USA; 4916 Olde Millcrest Court, Raleigh, NC 27609 USA

**Keywords:** FGF21, Non-human primate, Body weight, Obesity, K-PD, Energy balance, Mathematical Model

## Abstract

PF-05231023, a long-acting FGF21 analogue, is a promising potential pharmacotherapy for the treatment of obesity and associated comorbidities. Previous studies have shown the potential of FGF21 and FGF21-like compounds to decrease body weight in mice, non-human primates, and humans; the precise mechanisms of action remain unclear. In particular, there have been conflicting reports on the degree to which FGF21-induced weight loss in non-human primates is attributable to a decrease in food intake versus an increase in energy expenditure. Here, we present a semi-mechanistic mathematical model of energy balance and body composition developed from similar work in mice. This model links PF-05231023 administration and washout to changes in food intake, which in turn drives changes in body weight. The model is calibrated to and compared with recently published data from cynomolgus macaques treated with PF-05231023, demonstrating its accuracy in describing pharmacotherapy-induced weight loss in these animals. The results are consistent with the hypothesis that PF-05231023 decreases body weight in cynomolgus macaques solely by a reduction in food intake, with no direct effect on energy expenditure.

## Introduction

Type 2 diabetes mellitus (T2DM) is a substantial and growing global health burden [1, 2], and obesity is a major contributing factor to the development of T2DM [3–6]. There is, therefore, a need for novel pharmacotherapies for the treatment of obesity and its comorbidities. Since its initial identification [7], fibroblast growth factor 21 (FGF21) has been shown to be a key hormonal regulator of glucose metabolism and energy balance [8–11], representing a promising potential approach for the treatment of obesity and T2DM. PF-05231023 is a long-acting FGF21 analogue that has been shown to decrease body weight and improve glucose tolerance in rodents [12].

FGF21 is known to signal through the FGFR1c receptor and its cofactor *β*-klotho [9, 13] and appears to have both central and peripheral effects. In the periphery, adipose tissue is believed to be the primary target tissue, with adiponectin-mediated downstream effects in the liver [14–16]. FGF21 may also act on the leptin axis [17–19]. In the central nervous system (CNS), FGF21 may alter metabolism by affecting circadian rhythm [20]. In mice with CNS-specific knockout of *β*-klotho, FGF21 does not cause weight loss [21].

In spite of these findings, there remains considerable uncertainty regarding the FGF21 mechanism(s) of action *in vivo* [9]. In rodents, FGF21 has been observed to decrease body weight by increasing energy expenditure [12, 22–24]. In non-human primates (NHP), there are conflicting results: while an FGF21 analog reduced both body weight and food intake [8], a monoclonal antibody targeting the FGFR1c/*β*-klotho complex reduced body weight without significant changes in food intake [9]. Thus, it is an open question as to whether, or to what extent, FGF21-mediated changes in body weight are driven by changes in food intake and/or energy expenditure in NHP.

As FGF21 mimetics proceed into the clinic [25, 26], there is significant interest in understanding the in vivo mechanism(s) responsible for the observed therapeutic effects. In a recent study in non-human primates [26], multiple doses of PF-05231023 were tested for efficacy in reducing food intake and body weight in male cynomolgus macaques. To help determine the relative roles of decreased food intake and increased energy expenditure in contributing to the observed dose-responsive decrease in body weight, food intake was measured daily for each animal and body weight was measured weekly.

Based upon similarities in percent body weight change between pair-fed animals and the highest-dosed groups, it appears that a direct effect of PF-05231023 to decrease energy intake is sufficient to explain the observed decrease in body weight in NHP. However, there are several confounding factors. The pair-fed arm of the study was completed two months after the initiation of the dosing arms, so that temporal differences as well as differences in baseline characteristics could play a confounding role. As with all pair-feeding studies, subtle behavioral differences (e.g., a stress response caused by perceived food insecurity) could lead to an altered metabolic phenotype. In rats, changes in body composition associated with weight loss induced by caloric restriction appear to be distinct from changes in body composition induced by pharmacotherapy [27]. In NHP, food access insecurity has been linked to changes in body mass deposition [28–30].

To address these potentially confounding factors in a rigorous manner, a semi-mechanistic mathematical model of body weight regulation is proposed based upon energy balance principles. Similar models have been developed and validated for use with data from mice [31, 32], rats [27], and humans [33], including for pharmacotherapy intervention [27, 34, 35]. PF-05231023 action is linked to changes in food intake through an inferred PKPD model. The dose-responsive link between PF-05231023 administration and a hypothesized mechanism of action (in this case, food intake reduction) is used to demonstrate the NHP data are consistent with the hypothesis that PF-05231023 acts directly only via a reduction in food intake. By withholding one of the study groups from the model calibration procedure, the predictive capability of the model is assessed.

To our knowledge, this is the first demonstration of an energy balance model to analyze weight loss data in NHP in the presence of pharmacotherapy. The results demonstrate both the flexibility of the proposed modeling framework, as well as the power of mathematical modeling as a tool to support biological understanding.

## Methods

All animal studies were conducted in accordance with animal care and use protocols approved by the Institutional Animal Care and Use Committee (IACUC) of Pfizer, Inc. All procedures performed on any animals were in accordance with regulations and established guidelines and were reviewed and approved by a Pfizer Institutional Animal Care and Use Committee. All experiments within this manuscript were undertaken to minimize animal suffering during the experiment.

This study complied with all applicable sections of the Final Rules of the Animal Welfare Act regulations (Code of Federal Regulations, Title 9), the Public Health Service Policy on Humane Care and Use of Laboratory Animals from the Office of Laboratory Animal Welfare, and the Guide for the Care and Use of Laboratory Animals from the National Research Council. The protocol and any amendments or procedures involving the care or use of animals in this study were reviewed and approved by the Testing Facility Institutional Animal Care and Use Committee before the initiation of such procedures. All animals were monitored and routine physical checkups were conducted by veterinarians of the Testing Facility throughout the study period.

### Study design

The study protocol has been described in detail previously [26]. Here, key components are summarized that are relevant to the present analysis.

Untreated spontaneously obese male cynomolgus macaques were individually housed in stainless steel cages described in the Guide for the Care and Use of Laboratory Animals. Animals were chair-trained and acclimatized (including to vehicle injections) for three weeks prior to study start. Seven days prior to study start, 36 animals were randomized into 6 groups based upon body weight and fasting TG. Baseline animal characteristics are summarized in Table 1.

**Table 1:** Summary of baseline characteristics for each treatment group

	Group 1 (*n* = 5)	Group 2 (*n* = 6)	Group 3 (*n* = 6)	Group 4 (*n* = 6)	Group 5 (*n* = 6)	Group 6 (*n* = 6)
Age (yr)	13.8 ± 0.8	14.5 ± 0.5	14.0 ± 0.9	12.9 ± 1.1	13.1 ± 1.1	12.6 ± 1.3
BW_0_ (kg)	10.1 ± 0.9	9.9 ± 1.3	10.6 ± 1.0	10.4 ± 0.3	10.6 ± 0.8	11.0 ± 1.0
BMI (kg/m^2^)	51.4 ± 4.6	48.0 ± 5.5	51.3 ± 2.9	48.0 ± 0.9	54.0 ± 2.8	52.1 ± 3.4
CHOL (mg/dL)	94.5 ± 7.9	103.2 ± 7.4	94.7 ± 12.3	105.4 ± 6.8	99.8 ± 9.7	111.3 ± 10.5
TG (mg/dL)	104.2 ± 25.7	96.2 ± 22.3	177.8 ± 46.1	192.2 ± 48.6	119.3 ± 24.9	202.8 ± 85.2
GLU (mg/dL)	98.2 ± 11.8	86.4 ± 4.4	165.3 ± 29.4	113.6 ± 16.3	92.8 ± 15.1	79.7 ± 8.8
HDL (mg/dL)	44.8 ± 6.2	47.8 ± 4.3	42.5 ± 3.9	51.2 ± 6.5	47.4 ± 6.8	53.9 ± 7.7
LDL (mg/dL)	34.8 ± 7.9	41.4 ± 4.5	27.7 ± 7.0	29.1 ± 1.5	36.4 ± 3.6	33.8 ± 4.0

After the conclusion of the initial study, animals from Group 1 were pair-fed to the mean food intake of Groups 4–6

*BMI* body mass index;* CHOL* total cholesterol;* TG* plasma triglycerides;* GLU* plasma glucose;* HDL* plasma high-density lipoprotein;* LDL* plasma low-density lipoprotein. All data are reported as mean ± SE

Groups received intravenous injections twice per week for four weeks of vehicle (2 mL) or PF-05231023 (0.1, 1.0, or 10.0 mg/kg). Following the end of the four week treatment period, the vehicle, 0.1, and 1.0 mg/kg groups began receiving vehicle only. The 10.0 mg/kg group received either vehicle only, 5 mg/kg once weekly, or 5 mg/kg twice weekly. The treatment groups are summarized in Table 2.

**Table 2:** Summary of treatment for each group

Group	Treatment description
Group 1	Vehicle during treatment and washout phases (control group)
Group 2	0.1 mpk PF-05231023 twice/wk; vehicle only during washout period
Group 3	1.0 mpk PF-05231023 twice/wk; vehicle only during washout period
Group 4	10.0 mpk PF-05231023 twice/wk; vehicle only during washout period
Group 5	10.0 mpk PF-05231023 twice/wk; 5 mpk PF-05231023 once/wk during washout period
Group 6	10.0 mpk PF-05231023 twice/wk; 5 mpk PF-05231023 twice/wk during washout period

After the conclusion of the initial study, animals from Group 1 were pair-fed to the daily mean food intake of Groups 4–6

For this analysis, time zero coincides with the administration of the first dose. Thus, doses were administered at 0, 3, 7, 10, 14, 17, 21, 24, 28, 31, 35, 38, 42, and 45 days. At each dose time, all animals received an injection, with vehicle replacing PF-05231023 for animals not assigned to active dosing.

### Food intake data

Pelleted food was provided twice daily (morning and evening) for a period of 30 minutes. Food weight was measured before and after administration, with the difference (correcting for spillage) recorded as consumption. The pelleted food consisted of 180 g/kg protein, 36 g/kg fiber, 59 g/kg fat, 60 g/kg ash, 10.3 g/kg calcium, 7.8 g/kg phosphorous, and 94.8 g/kg moisture, with the remaining 552.1 g/kg assumed to be carbohydrate. Animals were also provided with 100 g of apple slices each day. Animals deemed to be sick or otherwise in need of veterinary intervention were occasionally given either a steamed cake or rice cereal. In both cases, the weight (steamed cake) or volume (rice cereal) was recorded. No veterinary interventions were associated with PF-05231023 administration. All animals were given ad libitum access to fresh drinking water for the duration of the study.

### Energy content of food

The caloric density of fat is assumed to be 9 kcal/g; the caloric densities of protein and carbohydrate are assumed to be 4 kcal/g. All other nutrients are assumed to provide negligible caloric content. Thus each gram of the pelleted food consists of 2.2084 kcal of carbohydrates, 0.72 kcal of protein, and 0.5310 kcal of fat, for a total caloric density of 3.4594 kcal/g. The caloric content of apples is assumed to be 0.52 kcal/g [36]. The steamed cakes are assumed to be 90 % carb with no other caloric content, and thus have an energy density of 3.6 kcal/g (4 kcal/g × 90 %). The rice cereal is assumed to have been mixed 1:1 (cereal:water) and thus have an energy density of 0.51 kcal/mL [37].

### Body weight data

Body weight for all animals was measured weekly prior to the administration of the first dose for that week.

### Data analysis

To minimize interindividual variation between study animals, all data are averaged within each group. Certain animals consumed all food administered during the baseline period, and thus could not be considered to be consuming *ad libitum*. Inclusion of these animals could lead to a biased analysis as a result of differences in perception of food security among the groups. Thus, these animals have been discarded from the present analysis (1 animal each from Groups 1, 2 and 4). As the groups are still approximately balanced, this exclusion does not diminish the findings of this analysis.

Each source of food intake (pelleted food, apples, steamed cake, rice cereal) is converted to kilocalories and the total caloric intake is computed for each animal for each day. Then mean caloric intake is computed for each group for each day of the study. Mean body weight data are computed for each group for each measurement time. All data are reported as mean ± standard error of the mean (SE).

### Body weight model

Previous authors have developed a mathematical model of body weight change in response to energy imbalance which has been used to describe dynamic body weight regulation in humans and mice [31, 32, 38]. For small changes in body weight, this model can be written as a linear differential equation describing changes in body weight (*BW*) in response to altered food intake (*I*),

1 $$\rho \frac{\textit{dBW}}{dt} = \left( I - I_{0}\right) - \epsilon \left( \textit{BW} - \textit{BW}_{0}\right) ,$$

where $\textit{BW}_{0}$ (g) is body weight at time *t* = 0 (days, d), $I_{0}$ (kcal/d) is baseline energy intake (the intake rate at which $\textit{BW}_{0}$ is maintained), *ρ* (kcal/g) is the approximate energy density of metabolic tissue adjusted for the cost of mass deposition, and *ε* (kcal/g/d) is a constant of proportionality relating body weight to energy expenditure. A detailed derivation of this model is included in the Appendix.

### Food intake model

Food intake is treated as a continuous variable and changes in food intake are assumed to be driven entirely by pharmacotherapy,

2 $$I = I(t) = (1 + r) I_{0} f(t),$$

where the function *f*(*t*) is given by

3 $$f(t) = 1 - \frac{I_{\text {max}} C(t)}{C(t) + IC_{50}}.$$

Here, $I_{\text {max}}$ is the maximum inhibitory effect of treatment and $IC_{50}$ is the level of drug concentration *C*(*t*) at which 50 % inhibition is obtained. The term $(1 + r)$ represents the tendency of untreated animals to gain body weight. (The simplified body weight model has a steady state $\textit{BW} = \textit{BW}_{0}$ when $I = I_{0}$, so the factor of $1+r$ describes any offset from this steady state.)

In the absence of a well understood pharmacokinetic model, a ‘K-PD’ [39] model can be used instead. Such a model replaces a mechanistic pharmacokinetic model with the simplest functional form that reliably recapitulates the observed effects of the drug. In order to describe the onset and washout time for the current drug, each dose is described by the Bateman function. That is, given doses at times $\{t_{i}\}$, the effective concentration of the drug at the site of action is modeled as

4 $$C(t) = \sum _{i} \frac{D_{i}k_{a}H(t>t_{i})}{k_{a} - k_{e}}\left( e^{-k_{e}(t - t_{i})} - e^{-k_{a}(t - t_{i})}\right) .$$

Here, $D_{i}$ is the dose (mg/kg) adminstered at time $t_{i}$ and *H*(*t*) is the Heaviside function. The volume of distribution is omited from the K-PD model, and it is assumed $IC_{50}$ has been non-dimensionalized to this unknown volume. The sum, indexed by *i*, is taken over the administered doses for each group (Table 2).

### Model solution

For a given set of model parameters (see below) and given the dosing times $\{t_{i}\}$ and the administered doses $\{D_{i}\}$ for each group, Eqs. (2)–(4) are computed exactly. The function *I*(*t*) and the initial body weight $\textit{BW}_{0}$ are then used to compute $\textit{BW}(t)$ by Eq. (1). For the pair-feeding experiment, the food intake data are inserted directly into (1) as a piece-wise constant function.

Because *I* is a continuous variable but actual food intake is measured daily, Eq. (2) is integrated to compute *total* food intake for day *k*,

5 $$T_{k} = \int _{k}^{k+1} I(t) dt.$$

All computer code was written in MATLAB 7.11.0 (Mathworks, Inc.). The integration of Eqs. (1) and (5) was computed using the MATLAB routine ode15s, a multi-step solver implementing a numerical differentiation formula. For the pair-feeding experiment, Eq. (1) was integrated in discrete one day intervals to avoid discontinuities associated with the use of a piece-wise constant function for food intake.

### Data fitting

Absent sufficient prior information, model parameters must be inferred from the available data. As the model parameters are not directly observable, this inference can be accomplished indirectly using an ordinary least squares (OLS) approach. Only data from Groups 1–5 are used to estimate model parameters, with Group 6 used to test the predictive capacity of the model. The body weight model parameters $\rho$, $\epsilon$, the food intake parameters $I_{0}$ and *r*, and the K-PD model parameters $k_{a}$, $k_{e}$, $I_{\text {max}}$, and $IC_{50}$ must be estimated. Additionally, it is assumed that each group may have a distinct rate of baseline food intake, so that five separate parameters $I_{0}^{j}$ must be estimated. All model parameters are estimated simultaneously.

Define $\theta = \{I_{0}^{1},I_{0}^{2},I_{0}^{3},I_{0}^{4},I_{0}^{5},r,k_{a},k_{e},I_{\text {max}},IC50,\rho ,\epsilon \}$. Let $\hat{I}_{k}^{j}$ represent the mean food intake measured for group *j* on day *k* and let $T_{k}^{j}(\theta )$ represent the model solution computed according to Eq. (5) for group *j* and given model parameters *θ*. Then the OLS error for food intake is

6 $$J_{FI}(\theta ) = \sum _{j,k} \left( \hat{I}_{k}^{j} - T_{k}^{j}(\theta )\right) ^{2}.$$

Similarly, let $\hat{BW}_{k}^{j}$ represent the mean body weight measured for group *j* on day *k* and let $\textit{BW}^{j}(\theta )$ represent the model solution computed according to Eq. (1) for group *j* and given model parameters *θ*. Then the OLS error for body weight is

7 $$J_{\textit{BW}}(\theta ) = \sum _{j,k} \left( \hat{\textit{BW}}_{k}^{j} - BW^{j}(t_{i})\right) ^{2}.$$

For both Eqs. (6) and (7), the sum is computed over all time points at which the appropriate measurement was taken, and for Groups 1–5 (including the pair-feeding data for Group 1).

The OLS parameter estimate is

8 $$\begin{aligned} \hat{\theta }_{\textit{OLS}} = \arg \min \left( \frac{J_{\textit{FI}}(\theta )}{w_{\textit{FI}}} + \frac{J_{\textit{BW}}(\theta )}{w_{\textit{BW}}}\right) . \end{aligned}$$

Thus, both the FI and BW data are fit simultaneously. Here, $w_{FI} = 1$ and $w_{\textit{BW}} = 0.01^{2}$ to approximately balance the relative contributions of the food intake and body weight components in the total squared error. The minimization (8) was computed by a trust-region algorithm as implemented in the MATLAB routine lsqnonlin. All components of the estimate $\hat{\theta }_{\textit{OLS}}$ are uniquely determinable from the available data (work not shown).

## Results

The best-fit parameter values are summarized in Table 3. Point estimates are computed without confidence intervals as a result of uncertainty regarding a suitable statistical model of the data (see below).

**Fig. 1:**
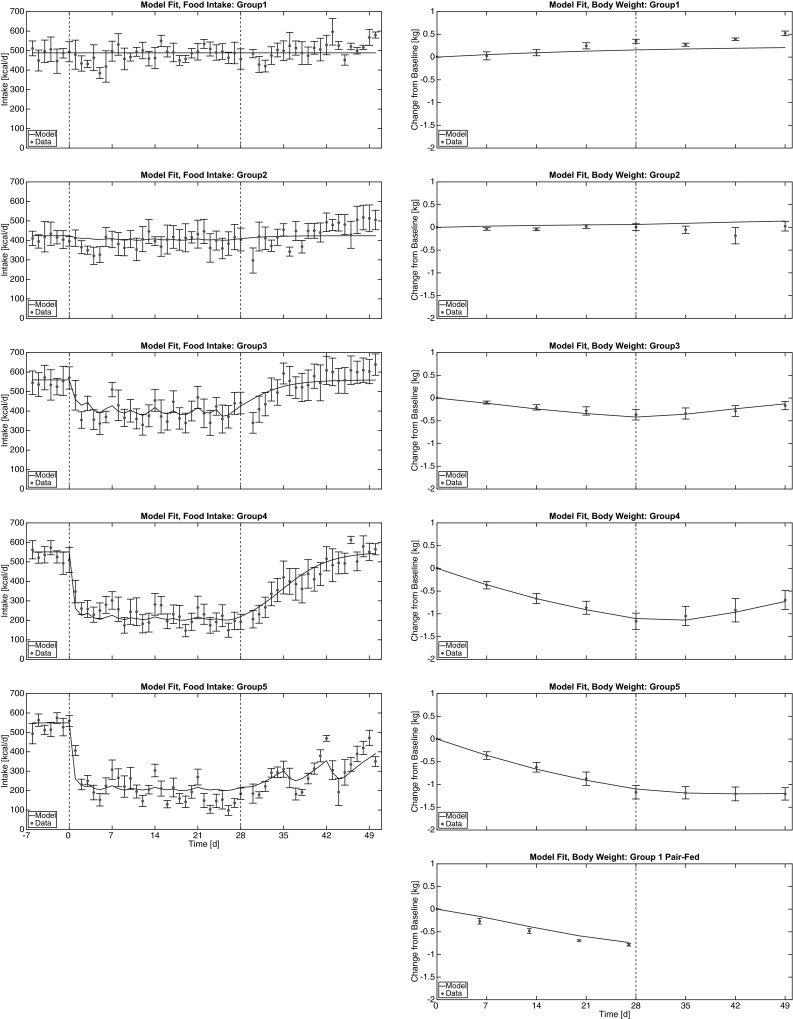
Model calibration. PF-05231023 is assumed to decrease food intake as described by the K-PD model (Eqs. (2)–(4)). This decrease in food intake drives a decrease in body weight according to Eq. (1). Best-fit model parameters were determined by OLS fit to the available data (Eq. 8). Model parameters are summarized in Table 3. *Left* Calibrated model solution for food intake, in comparison to experimental data. *Vertical dashed lines* indicate the start and end of the active-dosing period. *Right* Calibrated model solution for change in body weight, in comparison to experimental data. *Vertical dashed line* indicates the beginning of the washout period. Data are shown as mean ± SEM.

For two model parameters, nominal/theoretical values can be computed (see the Appendix) and compared to their OLS estimates. Interestingly, the estimates obtained using the calibration data set are not consistent with the theoretical values ($\rho = 8.4$ vs. estimated 4.81 kcal/g; $\epsilon = 0.05$ vs. estimated 0.162 kcal/g/d). This is most likely due to the fact that the theoretical values are consistent with changes in body composition which occur over long periods of time [38], but shorter term changes (e.g., alterations in fluid balance, glycogen stores, and protein metabolism) may alter these theoretical values. It is also possible that assumptions regarding the contributions of various mechanisms to energy expenditure [Eq. (11) in the Appendix] may be oversimplified. In spite of this potential shortcoming, there is no systematic modeling error between groups in the calibration data (Fig. 1) and, because the model parameters $\rho$ and $\epsilon$ are assumed to be the same for all groups, there is no evidence of dose-responsive changes in body composition or energy expenditure associated with PF-05231023 treatment.

**Table 3:** Summary of model parameters, nominal values, and OLS estimates. For parameters *ρ* and *ε*, theoretical values can be derived

Parameter	Description	Nominal value	Estimated value	Units
$I_{0}^{1}$	Baseline intake, Group 1	–	446.6	kcal/d
$I_{0}^{2}$	Baseline intake, Group 2	–	387.6	kcal/d
$I_{0}^{3}$	Baseline intake, Group 3	–	511.8	kcal/d
$I_{0}^{4}$	Baseline intake, Group 4	–	503.9	kcal/d
$I_{0}^{5}$	Baseline intake, Group 5	–	501.9	kcal/d
*r*	Overconsumption factor	–	0.094	–
$k_{a}$	Absorption/onset rate	–	2.03	d^−1^
$k_{e}$	Elimination/offset rate	–	0.26	d^−1^
$I_{\text {max}}$	Max inhibition of FI	–	0.71	–
$IC_{50}$	Concentration for 50 % inhibition	–	1.49	mg/kg
*ρ*	Mean whole-body energy density	8.4	4.81	kcal/g
*ε*	Energy expenditure coefficient	0.05	0.162	kcal/g/d

OLS estimates are obtained by fitting the parameters to the calibration data set according to Eq. (8). Because the K-PD framework is used to represent the onset and washout of PF-05231023, it is assumed that the parameter *IC*_50_ has been non-dimensionalized to the volume of distribution of the active metabolite at the site of action

The model solution corresponding to the best-fit parameter values is shown in comparison to the data in Fig. 1. The energy balance model describes the mean effect of PF-05231023 on food intake at all doses, including the washout period. This supports both the appropriateness of the K-PD framework as well as the dose-responsiveness of the hypothesized direct effect of PF-05231023 in reducing food intake. The modeled change in food intake leads to a predicted change in body weight that is consistent with the observed data at all measurement times, supporting the hypothesis that PF-05231023 acts to reduce body weight by decreasing food intake without directly altering energy expenditure [26].

**Fig. 2:**
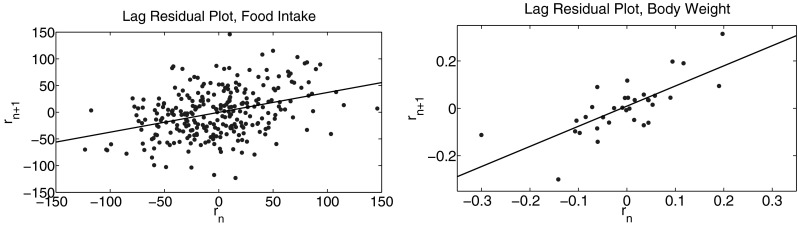
Statistically significant correlation between residuals at subsequent measurement times for food intake (*left* *p* = 4.5 × 10^−10^) and body weight (*right* *p* = 1.3 × 10^−6^).

The model residuals appear random and approximately normally distributed (Fig. 5 in the Appendix), as expected if the mathematical model is correctly specified [40]. However, the model solution appears to miss fluctuations in food intake that occur on a time scale shorter than one week (Fig. 1). This observation is supported by a lag residual plot (Fig. 2), demonstrating a statistically significant correlation between the modeling error at consecutive measurement times. This indicates that an ordinary least squares statistical model may not be appropriate [41] and confidence intervals for parameter estimates would be misleading [42]. However, it is not believed that a misspecified statistical model has a meaningful impact on the mean parameter estimates reported in Table 3 [42].

The discrepancy between the model and the data at time scales shorter than 7 days is likely caused by the complex pharmacology of PF-05231023, which has been hypothesized to operate at two distinct time scales in mice [12]. While the simple K-PD framework used in this study cannot represent such complex pharmacology, it does accurately describe the mean change in food intake over longer time periods for all doses in the calibration data set. As such, the proposed model is sufficient to draw conclusions regarding the relationship between PF-05231023, food intake, energy expenditure, and body weight change.

To further test the accuracy of the proposed energy balance model for NHP data following PF-05231023 treatment, the treatment protocol for Group 6 (10 mg/kg twice/wk for 4 weeks, followed by 5 mg/kg twice/wk) was simulated and compared to the collected data. Because data for Group 6 was not used in the model calibration, this provides a meaningful test of the accuracy of the model.

**Fig. 3:**
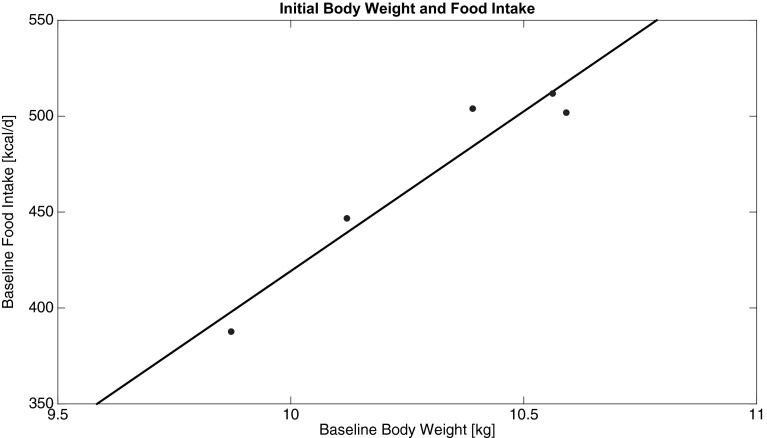
Relationship between estimated baseline food intake, *I*_0_, and body weight at *t* = 0, $BW_{0}$. Line of best fit: $I_{0} = 166.5 BW_{0} - 1246$ (p = 0.008).

For the calibration data sets, the baseline rate of energy intake, *I*_0_, was estimated for each group. The resulting parameter estimates exhibit an approximtely linear relationship with baseline body weight (Fig. 3). Thus, *I*_0_ for Group 6 could be extrapolated using the value of $BW_{0}$ for Group 6. This value was then used with the parameters from Table 3 to simulate the mathematical model.

**Fig. 4:**
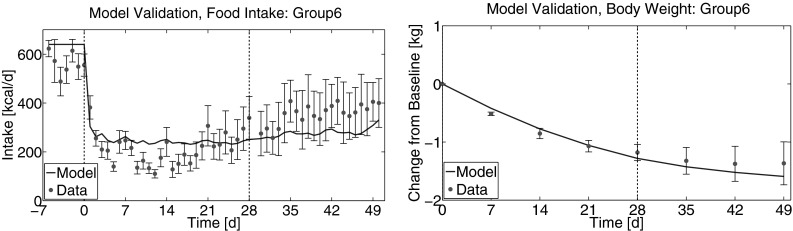
Assessment of model predictive capacity. Group 6 treatment protocol was simulated based upon model parameters as estimated from the calibration data set (Table 3). Baseline food intake was estimated based upon a linear relationship between $I_{0}$ and $BW_{0}$ observed in the calibration data set (Fig. 3) but no additional parameters were fit to data from Group 6. *Left* predicted food intake in comparison to data from Group 6. *Vertical dashed lines* indicate the start and end of the active-dosing period. *Right* predicted change in body weight in comparison to data from Group 6. *Vertical dashed line* indicates the beginning of the washout period. Data are shown as mean ± SEM.

**Fig. 5:**
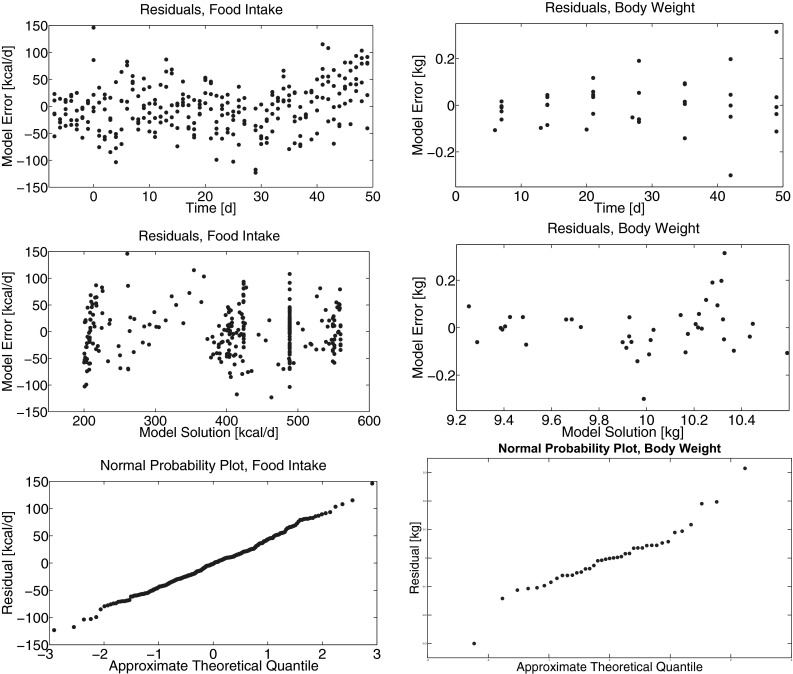
Residual plots for food intake (*left*) and body weight. Residuals do not exhibit any significant structure when viewed as a function of time (*top*) or model magnitude (*middle*), and appear to be approximately normally distributed (*bottom*), though there are two apparent outliers in the *BW* residuals.

Though the resulting estimate of *I*_0_ for Group 6 is slightly high, the simulation accurately estimates the mean change in food intake for Group 6, as well as mean change in body weight secondary to decreased food intake (Fig. 4). As for the calibration data set, fluctuations in food intake that occur on a time scale faster than 7 days are not well-modeled by the K-PD framework. The model over-predicts food intake during the first two weeks of treatment, but is accurate for weeks 3 and 4. Interestingly, the model is approximately constant during these earlier times, while food intake slowly increases from day 14 to day 28. No similar effect is observed in the calibration data, and the cause of this phenomenon in the Group 6 data is unknown. While the model does predict a slight increase in food intake after dosing is changed to 5 mg/kg at day 28, the change observed in the data is much larger. In spite of these shortcomings, the model provides an accurate prediction of mean food intake over the course of the experiment, and accurately predicts the change in body weight associated with the treatment. This is particularly true given the degree of variability exhibited in the data for Group 6, as seen in the magnitude of the error bars in Fig. 4 during the washout period.

## Discussion

Administration of PF-05231023 has been observed to be associated with dose-responsive decreases in food intake and body weight in NHP [26]. While pair-feeding data are strongly suggestive that PF-05231023 acts primarily to reduce food intake, the compound is known to cause increases in energy expenditure in mice [12]; moreover, there are conflicting reports regarding the effects on energy balance of other compounds targeting the FGF21 axis [8, 9]. While it is possible to measure energy expenditure directly, these data can be noisy and difficult to collect, and were not collected for this experiment. The present study is the first to our knowledge to develop an energy balance model for use in describing pharmacotherapy-induced weight loss in non-human primates. This model is used to analyze the degree to which decreases in food intake following treatment with PF-05231023 are responsible for the observed decreases in body weight in cynomolgus macaques.

The energy balance model linking food intake and energy expenditure to a change in body weight was adapted from a similar model originally developed for C57BL/6 mice [32]; comparable models have been used to describe dynamic body weight changes in humans [33] and rats [27]. These models have been shown to accurately describe changes in body weight and body composition following changes in food intake, and have even been used to describe and analyze pharmacotherapy-induced weight loss in mice [34] and humans [35]. However, it is possible that the broader metabolic effects of pharmacotherapy-induced weight loss are different from those associated with caloric restriction [27]. Thus pair-feeding data alone may not be sufficient to draw conclusions regarding the contributions of energy intake and energy expenditure to decreased body weight.

Here, a mathematical framework is established that describes changes in body weight associated with both PF-05231023 treatment and caloric restriction (pair-feeding) simultaneously. The only direct effect of PF-05231023 is assumed to be a reduction in food intake. The mathematical model does permit energy expenditure to change as a result of weight loss (i.e., because of the decrease in metabolically active body mass), but such changes occur identically in control, pair-fed, and actively-dosed animals. Thus these changes in energy expenditure depend only on existing (i.e., non-pharmacological) physiological mechanisms and *are not* associated with a direct response to treatment. This modeling framework accurately describes average food intake for all doses. The model also accurately describes changes in body weight for all doses, including for the pair-feeding arm of the current study. Thus any change in energy expenditure directly attributable to drug treatment must be within the modeling error, which is very small. As such, the model supports the hypothesis that PF-05231023 acts to reduce body weight in NHP strictly via reduction of food intake. The dose-responsiveness of this effect (as inferred via the K-PD formulation) further supports the hypothesis. Because the calibrated model is able to describe the rebound in food intake and body weight during the washout period, the reductions in food intake associated with PF-05231023 appear to be predominantly PK-driven.

Two of the model parameters inferred from the data, *ρ* and *ε*, can be compared to theoretical values. Interestingly, the values inferred differ significantly from the theoretical values (Table 3). These parameters represent, respectively, the average whole-body energy density (kcal/g) and the energy expenditure per unit body weight (kcal/g/d). There are several possible explanations for the discrepancy between the predicted and inferred values of these parameters.

First, the assumption that body composition can be broken down into fat mass and fat-free mass, each with its own (constant) energy density, may be too simple. This assumption ignores the presence of non-metabolic body mass such as extracellular fluid. While models similar to the one used here have been validated in both mice [32] and humans [33], these models are validated on the basis of their “long-term” behavior, so that changes occuring on faster time scales are not considered. Thus, for instance, slight changes in hydration that are distinct from changes in the metabolic components of fat and fat-free mass could bias the estimates of *ρ* and *ε*. It is also not necessary for all components of fat-free mass (e.g., glycogen vs protein stores) to change on the same time-scale, and such differences may be particularly noticeable in short-term studies. Indeed, in humans, components of body composition have been observed to change on different time scales [43], and a similar effect could be occuring here.

A second possibility involves limitations in the energy expenditure model itself. Numerous metabolic and hormonal changes are known to be associated with changes in energy balance. While the model does include body composition and adaptive thermogenesis as components of energy expenditure, it is possible that the representations of these quantities are oversimplified.

A third possibility is the existence of biases/tradeoffs associated with the simultaneous fitting of BW and FI data. For instance, the model is limited in its ability to describe fluctuations in food intake that occur with a period of 2–3 days. Yet, any such biases in the estimates of *ρ* and *ε* would have to be quite small given the accuracy of the fits to FI (over periods greater than 7 days) and BW data (Fig. 1). Moreover, food intake data was used directly as an input into the body weight model (as opposed to using the model-predicted food intake) for the pair-fed group, while the fits to the body weight data are comparable between the pair-fed and actively-dosed groups. An alternative approach is to use the theoretical values of *ρ* and *ε* to simulate the body weight model (using either the model-predicted food intake or the food intake data directly) and compare the resulting simulations to the body weight data, along the lines of a similar analysis in mice [32]. However, this approach does not accurately recapitulate the observed BW data for any group (not shown). Because this limitation applies identically to the pair-fed as well as all actively-dosed groups, the discrepancies in the parameters *ρ* and *ε* seem to reflect a limitation in the representation of NHP physiology, and not any effect associated with PF-05231023.

The energy balance model presented here is developed from the assumption that each unit of body mass gained or lost consists of some fixed fraction of fat versus fat-free mass. Physiologically, the regulation of macronutrient stores (and thus body composition) is a complex process depending upon the energy balance of an animal as well as many other factors (e.g., hormonal regulation). In male C57BL/6 mice, body composition has been shown empirically to be described by a time-invariant function which varies minimally between the animals in an experiment [31], though the relationship may be sexually dimorphic [34]. While the animals in the present study are not genetically identical, this assumption is expected to be sufficiently accurate for the present purpose (see Appendix).

**Fig. 6:**
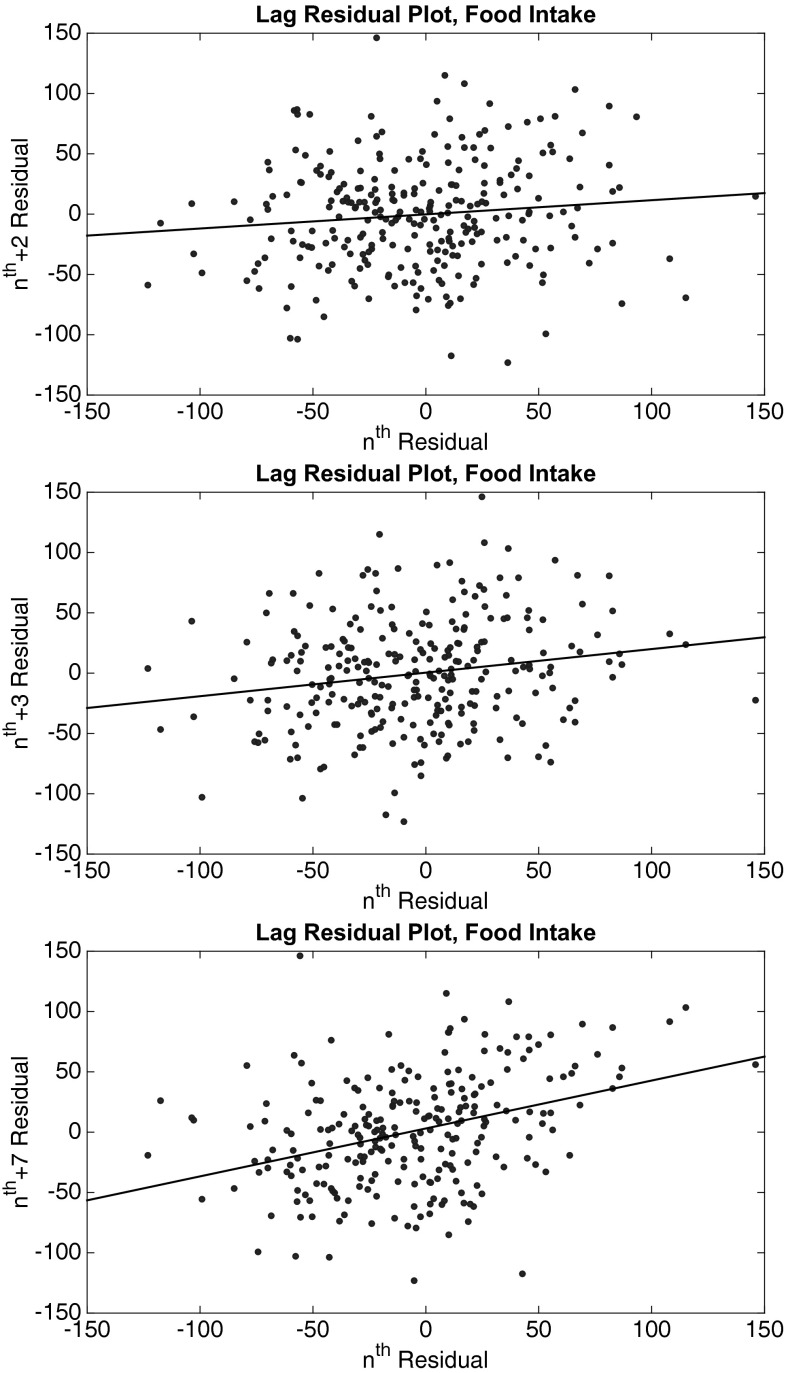
Correlation between food intake residuals. Statistically significant correlation between residuals separated by 1 day (Fig. 2), 3 days (*middle*; *p* = 0.0027), and 7 days (*bottom*; *p* = 8.1 × 10^−9^), but not 2 days (*top*; *p* = 0.065). Similarly, there is no statistically significant correlation between residuals separated by 4 or 5 days (not shown). This correlation structure is likely the result of the shortcoming of the K-PD model to describe the multiple time scales [12] on which PF-05231023 is hypothesized to act.

A statistically significant correlation structure is observed in the modeling errors for food intake measurements collected on consecutive days. A similar structure is observed for measurements separated by 3 or 7 days, but not two days (Fig. 6 in the Appendix). Because dosing was administered twice weekly on the first and fourth day of the week, it seems likely that the observed modeling error is associated with the shortcomings of the simple K-PD framework for describing the PF-05231023 mechanism of action. Conversely, the proposed mathematical model provides an accurate estimate of the mean food intake over periods greater than one week. In rodents, PF-05231023 has been proposed to operate at two different time scales as a result of different rates of clearance of the N- and C-terminal clippings of the original molecule [26]. The residual analysis presented here is consistent with a similar mechanism in NHP; the simple K-PD framework used to model the onset and washout of treatment accurately describes the longer-duration mechanism but not the shorter-duration mechanism. A more mechanistic PKPD model of PF-05231023 is complicated both by the complex dynamics of the molecule [26] as well as the mechanistic uncertainties surrounding FGF21 itself [9], and such work is beyond the scope of the current study. Crucially, the model presented here accurately describes the dose-responsive change in total food intake associated with PF-05231023 and supports the hypothesis that PF-05231023 decreases body weight only by its direct effect on food intake. The K-PD framework used here establishes a basis for model-based assessment of alternative doses and/or dose schedules in future studies.

As elsewhere [31, 32, 38, 44, 45], the body weight model is intended to describe changes in energy balance and body weight which occur over periods of time greater than one day. Dynamics which occur at a faster time scale (e.g., meal patterns within a day) are not represented. As such, no attempt is made to represent possible pharmacotherapeutic actions of PF-05231023 on metabolic endpoints such as plasma insulin and triglycerides, although such effects have been reported [9, 46]. It has been shown [38] that the energy balance framework used here is qualitatively consistent with a hypothetical model of within-day metabolism which has been averaged over a longer time period. The actual implementation of such a within-day model, and a quantitative comparison to the present results, is an interesting open problem for future work. Thus, there is significant scope for expansion of the analysis presented here as additional mechanistic details become available for PF-05231023 pharmacology and FGF21 mechanism(s) of action.

## Appendix: Body weight model derivation

Begin with the basic two-state model [31, 32, 38] which describes the relationship between fat mass ($\textit{FM}$) and fat-free mass ($\textit{FFM}$) during energy imbalance:

9 $$\begin{aligned} \rho _{\textit{FM}}\frac{\textit{dFM}}{\textit{dt}}&= \frac{\rho _{\textit{FM}}}{\rho _{\textit{FM}} + \alpha \rho _{\textit{FFM}}}\left( I - E\right) \nonumber \\ \rho _{\textit{FFM}}\frac{\textit{dFFM}}{\textit{dt}}&= \frac{\alpha \rho _{\textit{FFM}}}{\rho _{\textit{FM}} + \alpha \rho _{\textit{FFM}}}\left( I - E\right) . \end{aligned}$$

Here, $\textit{FM}$ and $\textit{FFM}$ represent stores of fat mass and fat-free mass (both measured in grams), with metabolic densities $\rho _{\textit{FM}}$ and $\rho _{\textit{FFM}}$ (kcal/g). *I* and *E* represent rates of energy intake and expenditure, both measured in kcal/d. The function *α* describes the manner in which body composition changes during energy imbalance,

10 $$\begin{aligned} \alpha = \frac{\textit{dFFM}}{\textit{dFM}}. \end{aligned}$$

Such a relationship has been demonstrated empirically in mice [31, 32] and humans [47]. Energy expenditure is assumed to vary with body composition and the rate of energy intake,

11 $$\begin{aligned} E&= K + \beta (I - I_{0}) + \lambda \textit{BW} + \gamma _{\textit{FM}}{} \textit{FM} + \gamma _{\textit{FFM}}{} \textit{FFM}\nonumber \\ {}&\quad + \eta _{\textit{FM}}\frac{\textit{dFM}}{\textit{dt}} + \eta _{\textit{FFM}}\frac{\textit{dFFM}}{\textit{dt}}. \end{aligned}$$

Here, $\beta$ (dimensionless) reflects the change in energy expenditure associated with changes in energy intake (diet-induced thermogenesis and adaptive thermogenesis; $\lambda$ (kcal/g/d) is the coefficient of physical activity, $\gamma _{\textit{FM}}$ and $\gamma _{\textit{FFM}}$ are proportionality constants relating basal metabolic rate to $\textit{FM}$ and $\textit{FFM}$, respectively; and $\eta _{\textit{FM}}$ and $\eta _{\textit{FFM}}$ (kcal/g) are the metabolic costs of mass deposition for fat mass and fat-free mass, respectively. All other energy expenditure mechanisms are assumed to be described by the zero-order term *K*. This model has been shown to accurately predict the dynamics of body weight change in mice [32], and a motivation for the individual terms in Eq. (11) can be found there. It should be noted that energy expenditure is modeled to change in response to changes in body weight and/or food intake, but no changes in energy expenditure are directly associated with pharmacotherapy.

In the absence of detailed, longitudinal body composition measurements, the function *α* is unknown. However, the function *α* can be reasonably approximated as a constant, and this approximation greatly simplifies the mathematical model. To show this, it is first shown that the mathematical model is, to first order, robust to the misspecification of the function *α*. (This permits the function *α* to be linearized.) Assume the energy partition function can be written as

12 $$\alpha (\textit{FM},\textit{FFM}) = \alpha _{0}(\textit{FM},\textit{FFM}) + \epsilon \alpha _{1}(\textit{FM},\textit{FFM}),$$

for some value of $\epsilon$, $|\epsilon |<<1$. Then

$$\begin{aligned} \frac{\textit{dFM}}{dt}&= \frac{I - E}{\rho _{\textit{FM}} + \rho _{\textit{FFM}}\alpha }\\ {}&= \frac{I - E}{(\rho _{\textit{FM}} + \rho _{\textit{FFM}}\alpha _{0}) + \rho _{\textit{FFM}}\epsilon \alpha _{1}}\\ {}&= \frac{\frac{I - E}{\rho _{\textit{FM}} + \rho _{\textit{FFM}}\alpha _{0}}}{1 + \frac{\rho _{\textit{FFM}}\epsilon \alpha _{1}}{\rho _{\textit{FM}} + \rho _{\textit{FFM}}\alpha _{0}}}\\ {}&\approx \frac{I - E}{\rho _{\textit{FM}} + \rho _{\textit{FFM}}\alpha _{0}} \left( 1 - \frac{\rho _{\textit{FFM}}\epsilon \alpha _{1}}{\rho _{\textit{FM}} + \rho _{\textit{FFM}}\alpha _{0}}\right) , \end{aligned}$$

where the final approximation follows from Taylor’s Theorem with the assumption

13 $$\begin{aligned} \left| \frac{\rho _{\textit{FFM}}\epsilon \alpha _{1}}{\rho _{\textit{FM}} + \rho _{\textit{FFM}}\alpha _{0}}\right| < 1. \end{aligned}$$

Similarly,

$$\begin{aligned} \frac{\textit{dFFM}}{\textit{dt}}&= \frac{\alpha (I - E)}{\rho _{\textit{FM}} + \rho _{\textit{FFM}}\alpha }\\ {}&= \frac{\alpha _{0}(I - E)}{\rho _{\textit{FM}} + \rho _{\textit{FFM}}(\alpha _{0} + \epsilon \alpha _{1})} + \frac{\epsilon \alpha _{1}(I - E)}{\rho _{\textit{FM}} + \rho _{\textit{FFM}}(\alpha _{0} + \epsilon \alpha _{1})}\\ {}&\approx \frac{\alpha _{0}(I - E)}{\rho _{\textit{FM}} + \rho _{\textit{FFM}}\alpha _{0}} \left( 1 - \frac{\rho _{\textit{FFM}}\epsilon \alpha _{1}}{\rho _{\textit{FM}} + \rho _{\textit{FFM}}\alpha _{0}}\right) . \end{aligned}$$

Thus the error resulting from a misspecification of the energy partition function accumulates at a rate which is no greater than first order in $\alpha$, and the approximation

$$\begin{aligned} \rho _{\textit{FM}}\frac{\textit{dFM}}{dt}&\approx \frac{\rho _{\textit{FM}}}{\rho _{\textit{FM}} + \rho _{\textit{FFM}}\alpha _{0}}(I - E)\\ \rho _{\textit{FFM}}\frac{\textit{dFFM}}{dt}&\approx \frac{\rho _{\textit{FFM}}\alpha _{0}}{\rho _{\textit{FM}} + \rho _{\textit{FFM}}\alpha _{0}}(I - E) \end{aligned}$$

is valid. It should be noted that the assumption (13) is not particularly restrictive. Given the values for $\rho _{\textit{FFM}}$, $\rho _{\textit{FM}}$, and $\alpha _{0}$ (see next section), the condition (13) is satisified if $\epsilon \alpha _{1} < 1.15$. Given the empirical value $\alpha _{0} \approx 0.77$ (next section), this means that the permissible (for the approximation to be valid) error in the function $\alpha$ can even exceed its nominal value.

Assume then, that $\alpha \approx \alpha _{0}$ is constant. At steady state,

14 $$I_{0} \approx E \approx K + \gamma _{\textit{FM}}{} \textit{FM}_{0} + \gamma _{\textit{FFM}}{} \textit{FFM}_{0} + \lambda (\textit{FM}_{0} + \textit{FFM}_{0}).$$

This expression can be solved for *K* and inserted into the sum of Eq. (9),

$$\begin{aligned} \rho _{\textit{FM}}\frac{\textit{dFM}}{\textit{dt}} + \rho _{\textit{FFM}}\frac{\textit{dFFM}}{\textit{dt}}&= I - E\\ {}&= \left( I - I_{0}\right) - \beta \left( I - I_{0}\right) - \left( \gamma _{\textit{FM}} + \lambda \right) \left( \textit{FM} - \textit{FM}_{0}\right) - \\ {}&{} \left( \gamma _{\textit{FFM}} + \lambda \right) \left( \textit{FFM} - \textit{FFM}_{0}\right) + \\ {}&{} \eta _{\textit{FM}}\frac{\textit{dFM}}{\textit{dt}} + \eta _{\textit{FFM}}\frac{\textit{dFFM}}{\textit{dt}}. \end{aligned}$$

By definition of $\alpha = \alpha _{0}$,

$$\frac{\textit{dFFM}}{\textit{dt}} = \alpha _{0}\frac{\textit{dFM}}{\textit{dt}},$$

and thus

$$\frac{\textit{dBW}}{\textit{dt}} = (1 + \alpha _{0})\frac{\textit{dFM}}{\textit{dt}}.$$

Similarly,

$$\begin{aligned} \textit{FFM} - \textit{FFM}_{0} \approx \alpha _{0} \left( \textit{FM} - \textit{FM}_{0}\right) . \end{aligned}$$

Combining these expressions,

$$\begin{aligned} \left( \frac{\rho _{\textit{FM}} + \eta _{\textit{FM}} + \alpha _{0}\rho _{\textit{FFM}} + \alpha _{0}\eta _{\textit{FFM}}}{1 + \alpha _{0}}\right)&\frac{\textit{dBW}}{\textit{dt}} = \\ {}&\left( 1 - \beta \right) \left( I - I_{0}\right) - \\ {}&\left( \frac{\gamma _{\textit{FM}} + \alpha _{0}\gamma _{\textit{FFM}}}{1 + \alpha _{0}} + \lambda \right) \left( \textit{BW} - \textit{BW}_{0}\right) . \end{aligned}$$

While this expression involves many unknown parameters, it is nothing more than an exponential model,

$$\begin{aligned} \rho \frac{\textit{dBW}}{\textit{dt}} = \left( I - I_{0}\right) - \epsilon \left( \textit{BW} - \textit{BW}_{0}\right) \end{aligned}$$

where

15 $$\begin{aligned} \rho&= \frac{\rho _{\textit{FM}} + \eta _{\textit{FM}} + \alpha _{0}\rho _{\textit{FFM}} + \alpha _{0}\eta _{\textit{FFM}}}{(1 + \alpha _{0})(1 - \beta )}\end{aligned}$$

16 $$\begin{aligned} \epsilon&= \frac{1}{1 - \beta }\left( \frac{\gamma _{\textit{FM}} + \alpha _{0}\gamma _{\textit{FFM}}}{1 + \alpha _{0}} + \lambda \right) . \end{aligned}$$

It should be noted that, while this simplification reduces the mathematical model to only two unique parameters, implicit within this model is the energy expenditure model (11) that describes changes in energy expenditure associated with changes in body weight or composition. That is, while PF-05231023 pharmacotherapy is assumed to have a direct effect *only* on food intake, this change in food intake will lead to a change in body weight, which in turn will lead to changes in energy expenditure..

Finally, it is worth noting that the derivation presented here relies upon the relationship between $I_{0}$, *K*, $\textit{FM}_{0}$, and $\textit{FFM}_{0}$ to eliminate the parameter *K*. An alternative approach would be to linearize the functions $\alpha$ and *E* around a steady state ($\textit{FM}_{0}$,$\textit{FFM}_{0}$) associated with a rate of intake $I_{0}$ [38]. It is trivial to show that the two derivations are equivalent.

## Nominal parameter values

Using Eqs. (15)–(16), theoretical values can be computed for $\rho$ and $\epsilon$. Based upon prior work [32], $\rho _{\textit{FM}} = 9.44$, $\rho _{\textit{FFM}} = 1.80$, $\eta _{\textit{FM}} = 0.18$, and $\eta _{\textit{FFM}} = 0.23$. These values are believed to be common across species. The value of $\beta$ has been estimated to range from 0.25 in human infants to 0.40 in rats, and we assume $\beta = 0.25$ here. To estimate the parameters $\gamma _{\textit{FM}}$ and $\gamma _{\textit{FFM}}$, we use Klieber’s Law as in [32]. Assuming $\gamma _{\textit{FM}} = 3.15$ kcal/kg/day and $\gamma _{\textit{FFM}} = 21.6$ kcal/kg/day for a 70 kg (lean) human male and a baseline body weight of 6.5 kg for a (lean) cynomolgus macaque, we obtain $\gamma _{\textit{FM}} \approx 5.7$ kcal/kg/d $= 0.0057$ kcal/g/d and $\gamma _{\textit{FFM}} \approx 39.1$ kcal/kg/d $= 0.0391$ kcal/g/d for cynomolgus macaques.

The parameters *λ* and *K* must be determined empirically. To do so, we begin with Eq. (14). A spontaneously obese cynomolgus macacque (10.6 kg, the mean for all animals in the current study) consumes approximately 500 kcal/d and will have a body fat percentage of approximately 30 % assuming a body condition score of 3.5 [48]. Thus

17 $$\begin{aligned} 500 = K + (0.0057)(0.3)(10600) + (0.0391)(0.7)(10600) + 10600\lambda . \end{aligned}$$

Physical activity expenditure (PAE) is defined to be the difference between total energy expenditure and resting metabolic rate, $\textit{PAE} = E - \textit{RMR} = \lambda \textit{BW}$. Also, for less active animals it is reasonable to assume a physical activity level (the ratio of total energy expenditure to resting metabolic rate; $\textit{PAL} = E/\textit{RMR}$) of 1.5 [49]. Thus

$$\lambda \textit{BW}_{0} = E - \textit{RMR} = (\textit{PAL} - 1)\textit{RMR} = 0.5\textit{RMR}.$$

Using the definition of resting metabolic rate, $\textit{RMR} = K + \gamma _{\textit{FM}} \textit{FM} + \gamma _{\textit{FFM}} \textit{FFM}$,

18 $$10600\lambda = 0.5\textit{RMR} = 0.5\left( K + (0.0057)(0.3)(10600) + (0.0391)(0.7)(10600)\right).$$

Solving the system of Eqs. (17)–(18) for *K* and $\lambda$ yields $K \approx 25$ kcal/d and $\lambda \approx 0.0157$ kcal/g/d.

The final unknown parameter is the body composition partition ratio, *α*. Based upon the data reported in [48] for male rhesus macaques,

$$\textit{FM} = -4.1262 + 0.5658\textit{BW} = -4.1262 + 0.5658(\textit{FM} + \textit{FFM}),$$

and thus

$$\textit{FFM} \approx 0.77 FM + 7.3.$$

Assuming that this cross-sectional relationship is indicative of the dynamic relationship between *FM* and *FFM*, these data would imply *α* = 0.77 for rhesus macaques, and we will use this value for cynomolgus macaques as well. Inserting all of these values in Eqs. (15)–(16) yields $\rho \approx 8.4$ and $\epsilon \approx 0.05$.

## Residual plots

Figures 5 and 6.
